# The fungal-derived compound AM3 modulates pro-inflammatory cytokine production and skews the differentiation of human monocytes

**DOI:** 10.3389/fimmu.2023.1165683

**Published:** 2023-10-09

**Authors:** Büsra Geckin, Gizem Kilic, Priya A. Debisarun, Konstantin Föhse, Azahara Rodríguez-Luna, Pablo Fernández-González, Ana López Sánchez, Jorge Domínguez-Andrés

**Affiliations:** ^1^ Department of Internal Medicine and Radboud Center for Infectious Diseases, Radboud University Medical Center, Nijmegen, Netherlands; ^2^ Radboud Institute for Molecular Life Sciences, Radboud University Medical Center, Nijmegen, Netherlands; ^3^ Cantabria Labs, Innovation and Development, Madrid, Spain; ^4^ Servicio de Dermatología, Hospital Universitario Ramon y Cajal, IRYCIS, Madrid, Spain

**Keywords:** immune response, homeostasis, cytokines, inflammation, AM3, disease, monocytes, macrophages

## Abstract

The proper functioning of the immune system depends on an appropriate balance between pro-inflammation and anti-inflammation. When the balance is disrupted and the system is excessively biased towards inflammation, immune responses cannot return within the normal range, which favors the onset of diseases of autoimmune or inflammatory nature. In this scenario, it is fundamental to find new compounds that can help restore this balance and contribute to the normal functioning of the immune system in humans. Here, we show the properties of a fungal compound with a strong safety profile in humans, AM3, as an immunomodulatory molecule to decrease excessive cytokine production in human cells. Our results present that AM3 treatment of human peripheral blood mononuclear cells and monocytes decreased their pro-inflammatory cytokine secretion following the challenge with bacterial lipopolysaccharide. Additionally, AM3 skewed the differentiation profile of human monocytes to macrophages towards a non-inflammatory phenotype without inducing tolerance, meaning these cells kept their capacity to respond to different stimuli. These effects were similar in young and elderly individuals. Thus, the fungal compound, AM3 may help reduce excessive immune activation in inflammatory conditions and keep the immune responses within a normal homeostatic range, regardless of the age of the individual.

## Introduction

Inflammation is a key component of immune responses. It promotes the mobilization of immune cells to the sites of infection or damage to eliminate the triggering factor, repair tissue damage, and restore homeostasis in the organism (1). Cytokines are molecules responsible for long-range intercellular communication and play a fundamental role as mediators during inflammatory processes. The actions of pro-inflammatory cytokines, such as interleukin (IL)-1, IL-6, or tumor necrosis factor-alpha (TNFα), need to be balanced with anti-inflammatory cytokines, such as IL-10 or IL-1 receptor antagonist (IL-1Ra). The latter contributes to the resolution of inflammation and the suppression of excessive immune activation, thus limiting the duration and the extent of the inflammatory responses (2). However, when there is an imbalance between these processes and the system is excessively biased towards inflammation, the immune responses cannot return within the normal range. This increases the risk of autoimmune and inflammatory conditions, such as cardiovascular diseases, inflammatory bowel disease, type II diabetes, or rheumatoid arthritis, among many others (3, 4). The globalization of the Western-type diet, a sedentary lifestyle, and the increasingly aging population led to a rise in the incidence and prevalence of diseases with an inflammatory component at an alarming rate. At present, inflammatory diseases are the leading cause of morbidity and mortality worldwide (5). This situation drives researchers from public and private institutions to find new compounds to tackle the inflammatory origin of these diseases.

Among the several available options, compounds of fungal origin are considered one of the most promising sources for molecules with immunomodulatory properties (6). In this respect, molecules derived from the cell wall of different species of fungi, such as β-glucans, zymosan, or mannans, are in the spotlight of many research projects for their possible use as (prophylactic) therapy against different types of diseases (7).

The active principle of Immunoferon, AM3, is a glycoconjugate compound renowned for its immunomodulatory effects (8, 9). This compound is produced by combining a naturally phosphorylated alpha glucomannan derived from the cell wall of a specific *Candida utilis* strain and soybean seed storage proteins. The polysaccharide segment is characterized by phosphoglucomannan-type α-glucan (roughly 150 kDa) with recurring polysaccharide linear chains comprised of α(1→6) and α(1→2) linkages among the mannose and glucose residues. Throughout its commercialization spanning more than four decades, the AM3 compound has been meticulously characterized at different levels, covering aspects such as molecular structure (10), bioavailability (11), metabolic processing (12), and clinical implications.

Multiple research works have shown the strong immunomodulatory properties of different molecules of fungal origin in innate immune cells, namely in monocytes/macrophages. In this regard, β-glucan derived from *Candida albicans* induces innate immune memory in monocytes and increases the production of inflammatory cytokines in response to a secondary stimulus (13, 14). Besides β-glucan, other fungal-derived compounds such as curdlan, yeast-b, and zymosan re-polarize macrophages towards a pro-inflammatory phenotype, with enhanced expression of CCR7, ICAM1, and CD80, and secretion of TNF-α and IL-6 (15).

We then hypothesized that AM3 may exert similar immunomodulatory activity on innate immunity cells. In this study, we describe how AM3 decreases exacerbated cytokine production by human monocytes. We show that AM3 skews the differentiation profile of macrophages towards a phenotype without pro-inflammatory features. Additionally, we show that the cell response to AM3 pre-treatment is not associated with the acquisition of immune tolerance.

## Methods

### Lactate dehydrogenase assay

The concentrations of AM3 used in this study were optimized in LDH assay (Promega, cat #G1780) to minimize cytotoxicity. Typically, adherent human peripheral blood mononuclear cells (PBMCs) obtained from healthy donors (Sanquin Blood Bank, Nijmegen, the Netherlands) were stimulated with different doses of AM3 (50 μg/mL, 10 μg/mL, 1 μg/mL and 0.1 μg/mL) for 24 hours. The AM3 compound (dissolved in water and sterilized by microfiltration through 0.22 µm cellulose acetate filters) was kindly provided by Cantabria Labs, Madrid, Spain. Collected supernatants were immediately used to perform the assay according to the manufacturer’s instructions.

### Different doses of AM3 *in vitro* tolerance and innate immune memory experiments

After written informed consent, buffy coats were obtained from healthy donors (Sanquin Blood Bank, Nijmegen, the Netherlands). Samples were anonymized to safeguard donor privacy. By differential density centrifugation, PBMC isolation was performed using Ficoll‐Paque (GE Healthcare, cat # GE17-1440-02). Cells were resuspended in Dutch modified RPMI 1640 medium (Invitrogen, cat #22409031) containing 50 µg/mL Gentamicin (Centrafarm), 1 mM Sodium-Pyruvate (Thermo Fisher Scientific, cat #11360088), 2 mM Glutamax (Thermo Fisher Scientific, cat #35050087) [base medium] and counted via Sysmex XN-450.

#### Tolerance experiments

PBMCs (4x10^5^ cells/well) were seeded in sterile flat-bottom 96-well tissue culture-treated plates (VWR, cat #734-2184) in the base medium and rested for an hour in a 37°C incubator supplied with 5% CO_2_ for monocytes to adhere. The cells were washed with warm PBS to eliminate excessive leukocytes and non-adherent cells in the wells. RPMI medium (used as a control condition), AM3 (0.1-50 μg/mL) or LPS (0.5 ng/mL, serotype O55:B5; Sigma-Aldrich, cat #L2880, further purified as described in (16)) was added on to monocytes (Day 0) and incubated for 24 hours in base medium supplemented with 10% human pooled serum. On Day 1, the cells were washed again with warm PBS and, thereafter, RPMI was replenished in the control group, while LPS was added to the AM3 group, and AM3 to the LPS group, followed by additional 24-hours incubation. At 48 hours (Day 2), cells were washed with warm PBS, resuspended in base medium with 10% human pooled serum, and incubated at 37°C, 5% CO2 for additional 4 days. On Day 6, exhausted medium was discarded and cells were restimulated with LPS (10 ng/mL) for the final 24 hours of the culture. Supernatants for cytokine measurements were collected on Days 1, 2, 6, and 7 and stored at -20°C. Secreted pro-inflammatory cytokines TNF-α, IL-6, and IL-1β and anti-inflammatory cytokines IL-10 and IL-1Ra levels were measured from supernatants after LPS restimulation using R&D Systems ELISA kits, following the manufacturer’s instructions. The results are presented in **Supplementary Figure 2**.

#### Innate immune memory experiments

PBMCs (5x10^5^ cells/well) were seeded in wells and adherent cells were enriched as described above. RPMI medium (negative control), AM3 (0.1-50 μg/mL), β-glucan (BG, 1 μg/mL), and Bacillus Calmette-Guérin (BCG 5 μg/mL *M. bovis*, BCG Denmark, AJ Vaccines) were added to the cells in base medium supplemented with 10% human pooled serum. After 24 hours (Day 1), wells were washed to remove any primary stimuli and fresh base medium supplemented with 10% human pooled serum was replenished to allow cells to eventually differentiate further (17). The cells were restimulated with LPS (10 ng/mL) as a non-related secondary stimulus on Day 6. After 24 hours of restimulation, supernatants were collected and stored at -20°C. Secreted pro-inflammatory cytokines TNF-α, IL-6, and IL-1β levels were measured from supernatants after restimulation using R&D Systems ELISA kits, following the manufacturer’s instructions. The results are presented in **Supplementary Figure 3**.

### Study approvals

Healthy donor samples were obtained from the Sanquin Blood Bank (Nijmegen, the Netherlands) after written informed consent. Sanquin Blood Bank is a commercial provider of blood products; as such, anonymized samples came without any specific ethics reference number or code. PBMCs from elderly and young donors (**Table 1**) were obtained from the clinical trials BCG Booster (NL58219.091.16) and BCG PRIME (NCT04537663). These samples were used to perform *in vitro* tolerance experiments to analyze difference in responsiveness between the PBMCs from elderly and young donors. The details of the procedure are as depicted above in the section “Different doses of AM3 *in vitro* tolerance and innate immune memory experiments”.

**Table 1:** Demographic data of elderly and young donors.

Demographics	*Elderly*	*Young*
Female, n	3	5
Male, n	6	4
Age, years	60-80	19-26
Body mass index	27.9 ± 5.1	23.6 ± 2.7

None of the donors have a history of BCG vaccination.

### Macrophage differentiation experiments

The samples were obtained from buffy coats, as explained above. Cells were resuspended in the base medium and counted via Sysmex XN-450. The cells (30x10^6^ for the AM3 group and 15x10^6^ for M-CSF and GM-CSF groups) were seeded into 60 mm tissue culture dishes (Corning, cat #430166) and rested for an hour in a 37°C incubator supplied with 5% CO_2_ to ensure monocyte adherence. Afterwards, non-adherent cells were removed by washing with warm PBS, and then, differentiation media were added to the cells, respectively as AM3 (10 μg/mL), M-CSF (10 ng/mL), and GM-CSF (10 ng/mL). After 24 hours, the cells of all the conditions were washed to mimic the innate memory/tolerance condition, and a base medium supplemented with 10% human pooled serum was added to the cells. On Day 3, culture media was removed and replenished with base medium supplemented with 10% human pooled serum with the corresponding differentiation factor, for M-CSF and GM-CSF conditions. On Day 6, supernatants were discarded, and cells were detached with cold PBS containing 5 mM EDTA, by gentle pipetting (6-10 times) around the dish surface before harvesting cell suspensions. This procedure was repeated three times to maximize cell-recovery as needed in flow cytometric analysis. Afterwards, cells were counted with a hematocytometer.

### Flow cytometry staining and analysis

Cells were resuspended in PBS for live/dead staining with 1:1000 diluted Fixable Viability Stain (FVS) 620-ECD (BD Biosciences, cat #564996) for 15 minutes at room temperature (RT). After a wash to remove the live/dead staining solution, the cells were resuspended in a 50 μL antibody panel cocktail (**Table 2**) prepared in PBS with 1% BSA and Brilliant Stain Buffer (BD Biosciences, cat #563794), and incubated for 45 minutes in the dark at RT. Thereafter, the antibody excess was washed and the cells were analyzed on the Cytoflex flow cytometer (Beckman Coulter).

**Table 2:** Macrophage differentiation flow cytometry panel.

Antibody-label	Channel	Dilution	Company	Cat #
CD68-APC	APC	1:20	Biolegend	333809
CD80-BV421	PB450	1:20	BD Biosciences	566263
CD86-PECy7	PC7	1:20	BD Biosciences	561128
CD163-FITC	FITC	1:20	Biolegend	333617
CD206-PE	PE	1:20	Biolegend	321105

### Metabolic assay

Lactate was measured from 24-hour, 48 hours, and 6 day supernatants of the tolerance experiment through a commercial fluorimetric assay. A standard curve was established by serially diluting 40 μM Na-L-Lactate (Sigma-Aldrich, cat# L7022). The samples and standards were placed into a 96-well flat-bottom black assay plate (Corning, cat# CLS3925). Afterwards, the reaction mix containing 100μM Amplex Red (Invitrogen, cat# A12222), 0.2 U/mL Horseradish Peroxidase (Thermo Scientific, cat# 31490), 2 U/mL Lactate oxidase (Sigma-Aldrich, cat# L9795) in PBS was added onto sufficiently diluted samples and standards. After 20 minutes of incubation at RT on a shaker, the fluorescence was measured with 530±25 and 590±35 filters (Biotek, SynergyHTX).

### Statistical analysis

GraphPad Prism 8 was used for all the statistical analyses. Three or more paired groups were compared using the Friedman test with Dunn’s multiple comparisons. Outcomes between two independent groups were analyzed by the Mann-Whitnney test used. A P-value of less than or equal to 0.05 was considered statistically significant. *: p ≤ 0.05, **: p ≤ 0.01, ***: p ≤ 0.001, ****: p ≤ 0.0001.

## Results

### AM3 decreases exacerbated pro-inflammatory cytokine secretion in human cells

We first sought to establish the ideal concentration of AM3 in our model, so we evaluated the cytotoxicity caused by four different concentrations of AM3 (50, 10, 1 and 0.1 μg/mL) to ensure we covered the full spectrum of activity. The LDH assay showed no cytotoxicity after 24 hours in any of the AM3 concentrations used (**Figure S1**); therefore, we decided to continue the experiments with the 10 µg/ml concentration leading to the lowest LDH production.

**Figure 1:**
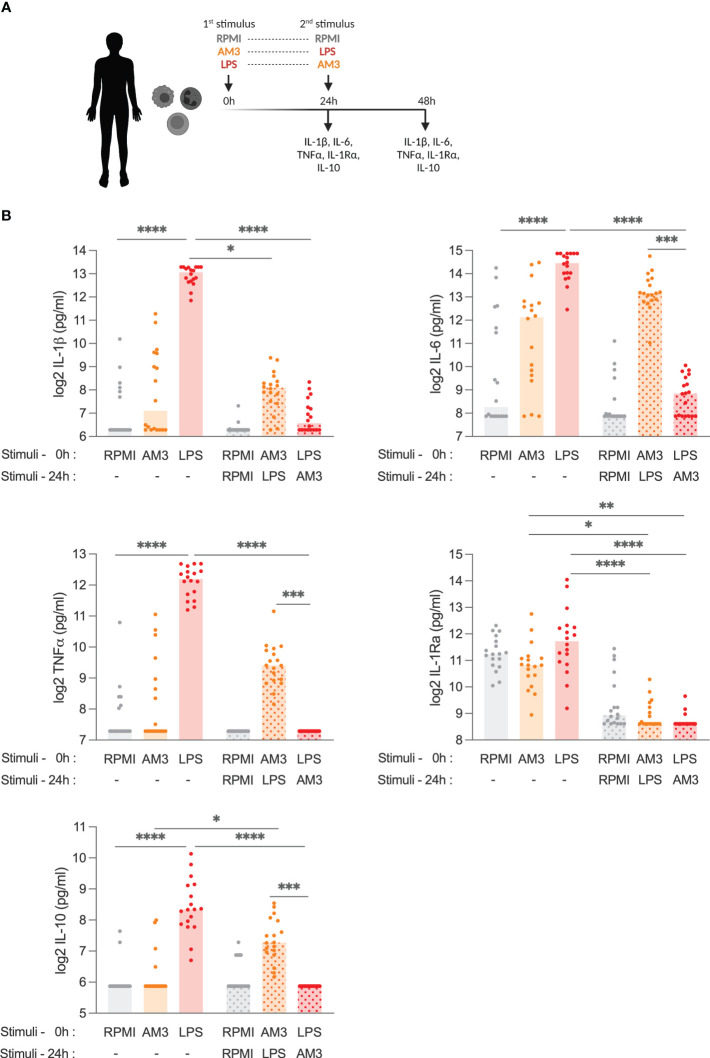
AM3 treatment limits LPS-driven cytokine response in human PBMCs **(A)** Experimental design: adherent PBMCs were stimulated with primary stimuli and supernatants were collected for cytokine detection at 24h, then cells were washed with control medium to remove any residue of the primary stimulation before secondary stimuli were added to the cultures following which the supernatants collected after an additional 24h (48h in total). **(B)** Cytokine production from PBMCs at 24 hours and at 48 hours after stimulation with medium control (RPMI), AM3 and LPS, eventually combined as by **(A)** in a number of healthy donors (n=18). Friedman test with Dunn’s multiple comparison was applied to compare experimental groups, *p<0.05, **p<0.01, ***p<0.001, ****p<0.0001. Bars without pattern: cytokine levels measured 24 hours after first stimulation, Patterned bars: cytokine levels measured 48 hours after first stimulation. Values for the groups RPMI (1st) + AM3 (2nd), RPMI (1st) + LPS (2nd), AM3 (1st) + RPMI (2nd), and LPS (1st) + RPMI (2nd) are presented in **Figure S2B**.

Next, we investigated the potential effects of AM3 on the cytokine production capacity of human PBMCs. Different from lipopolysaccharide (LPS), which induces robust cytokine production by human cells (18, 19), AM3 did not significantly increase pro-inflammatory (IL-1β, IL-6, and TNFα) or the anti-inflammatory (IL-1Ra or IL-10) cytokines, compared to control group (RPMI) (**Figures 1A, B**). Apart from non-significant trends in IL-6 production, there was no apparent change in cytokine production upon direct exposure to AM3, thus we tested whether AM3 treatment could modulate cytokine response in combination with (i.e., before and after) LPS stimulation. As expected, the stimulation with LPS strongly induced the production of the pro-inflammatory cytokines IL-1β, IL-6, and TNFα, and also that of anti-inflammatory IL-1Ra or IL-10, which are concomitantly produced as compensation to avoid excessive inflammation (**Figure 1B**). However, pre-treatment of human PBMCs with AM3, 24 hours prior to stimulation with LPS (AM3-LPS group), considerably mitigated the LPS-induced production of cytokines (**Figures 1B**; **S2B**). This suggests that AM3 exerts an immunomodulatory effect on LPS-stimulated cells. However, this decrease was significant only in the case of IL-1β and IL-1Ra. Upon this observation, we checked the ratio between IL-1β/IL-1Ra, further confirming our observations on AM3 immunomodulatory effects as all AM3 groups yielded in a ratio smaller than 1 (**Figure S2A**). Lower cytokine responses were observed upon AM3 treatment after LPS stimulation (LPS-AM3 group), however this was not dissimilar from the media restimulation controls (**Figure S2B**).

Conversely, there was no significant change in cytokine production when combined treatments (AM3-LPS and LPS-AM3) were compared to the AM3 group, except for IL-1Ra, which decreased evidently in any combination treatments. Additionally, different doses of AM3 in these settings did not yield to similar significant results (**Figure S3**).

### AM3 is similarly effective in the cells from both young and elderly individuals

Elderly individuals tend to be vulnerable to immune-related diseases and syndromes due to imbalanced immune responses during aging (20). One of the well-known reasons is that elderly people present a dysfunctional adaptive immune response (21), making them less responsive to vaccination and more susceptible to infections. Another reason is that elderly people tend to develop chronic inflammation (so-called “inflammaging”) (22).

**Figure 2:**
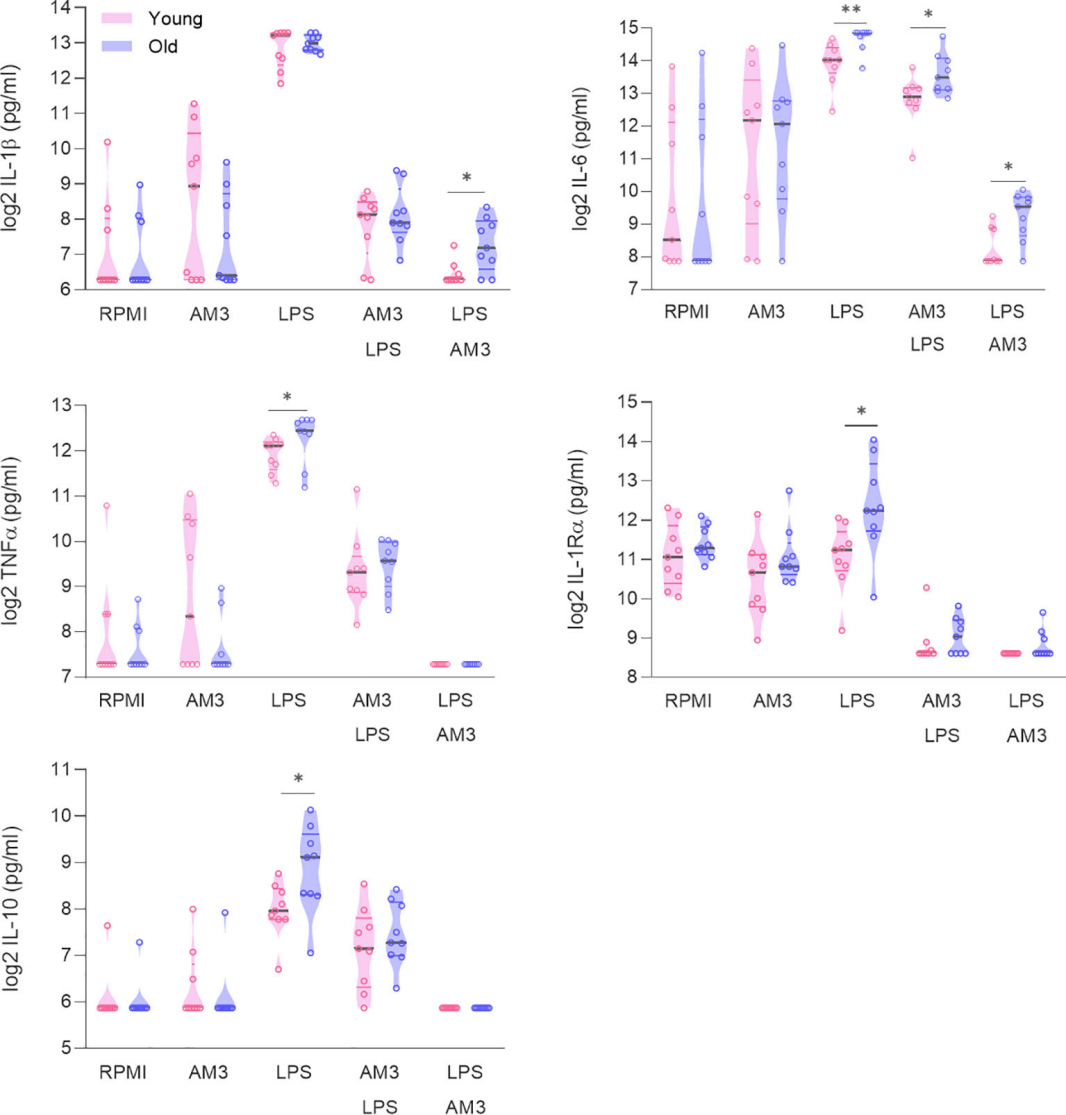
AM3 is similarly effective in cells from both young and elderly individuals. Cytokine production measured in supernatants from PBMCs after 24 hours and after 48 hours following primary stimulation (RPMI, AM3 and LPS) and combination treatments, in cultures derived from young (n=9, pink data points) and elderly (n=9, purple data points) healthy donors. Mann-Whitney test used to compare elderly and young individuals. *p<0.05, **p<0.01. The gray line depicts the median whereas colored lines represent the borders of interquartile range.

In this context, we hypothesized that the immunomodulatory effects of AM3 may help control dysregulated responses seen in aged individuals. Accordingly, we performed similar experiments comparing the responses to AM3 and LPS stimulation in cells obtained from young and aged (>60 years old) donors. Interestingly, we observed that when exposed to LPS, cells obtained from aged individuals produced significantly higher amounts of IL-6, TNFα, IL-1Ra, and IL-10 than those from younger donors (**Figure 2**). Treatment of the cells with AM3 prior to the challenge with LPS (AM3-LPS group) significantly decreased the induction of any of the cytokines studied. Furthermore, AM3 treatment maintained low cytokine responses after LPS exposure and cytokine levels equalized between the two age groups after AM3 treatment (LPS-AM3), except for IL-6 and IL-1β.

### AM3 skews the differentiation process of monocytes towards a non-inflammatory phenotype

**Figure 3:**
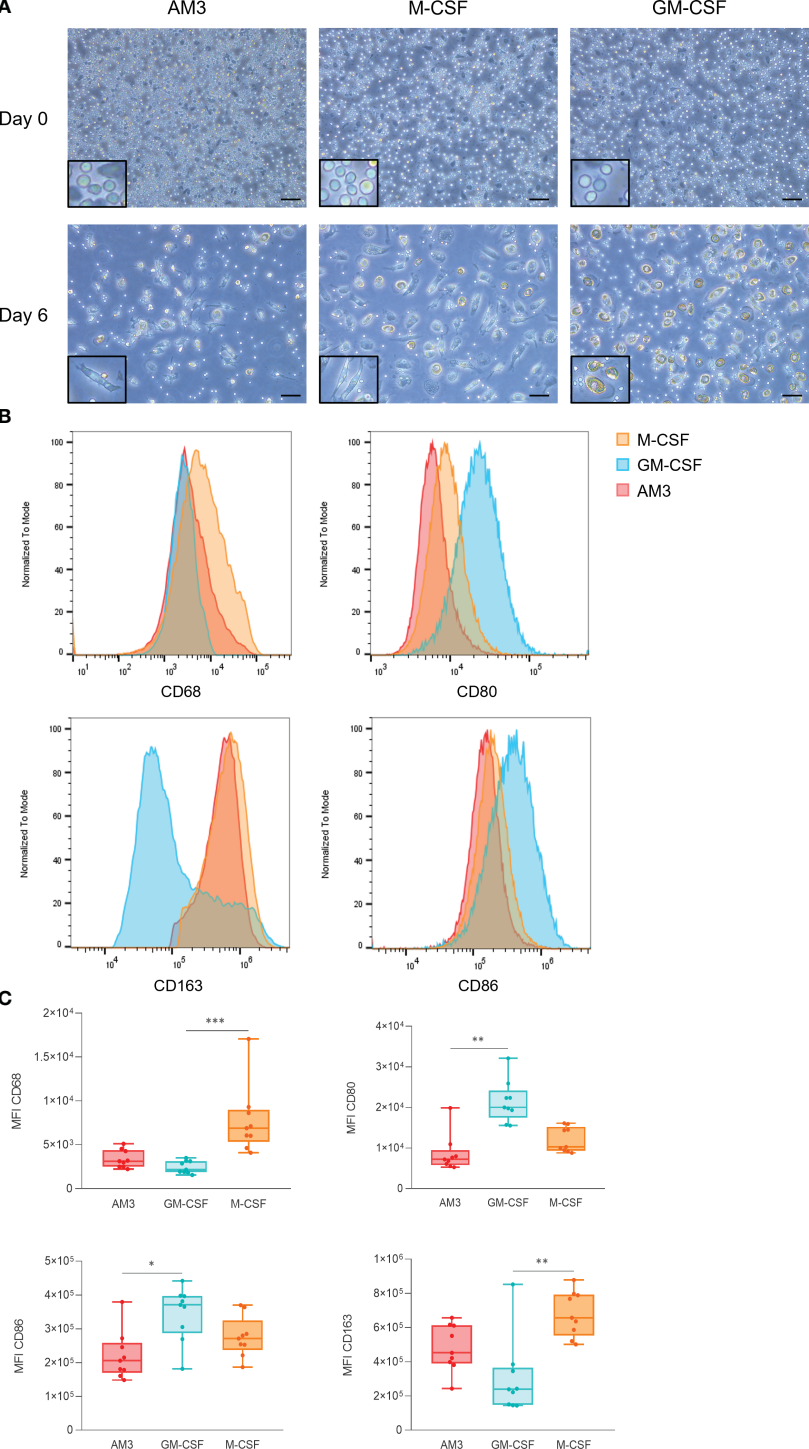
AM3 treatment of human monocytes skews the differentiation process towards an anti-inflammatory phenotype **(A)** Microscope images of cells at day 0 and day 6 of the differentiation process, magnification: 20X, scale bar: 100 µM. The areas highlighted in a black rectangle show an area of the same image with a larger magnification to better appreciate the cell morphology. **(B)** Flow cytometry analysis of macrophage markers at day 6 after exposure to AM3, M-CSF or GM-CSF. **(C)** Comparisons of MFIs by marker (CD68, CD80, CD86, and CD163) are depicted on the charts. n=9, Friedman test with Dunn’s multiple comparisons. *p<0.05, **p<0.01, ***p<0.001. For **(B)** and **(C)** all cells were gated as CD45+ CD206+ live cells.

The effects of AM3 on human PBMCs presented so far suggest an anti-inflammatory effect that limits LPS-induced cytokine production, regardless of the donor’s age. These results led to the hypothesis that AM3 might play a role in skewing the differentiation of monocytes to macrophages towards an anti-inflammatory phenotype. To test this, we obtained monocytes from the peripheral blood of healthy donors and subjected them to well-established monocyte-to-macrophage differentiation protocols in the presence of granulocyte-macrophage colony-stimulating factor (GM-CSF) and macrophage colony-stimulating factor (M-CSF), respectively promoting pro-inflammatory and anti-inflammatory macrophages, in parallel to AM3. We then assessed phenotypic characteristics in these cultures and found that AM3-macrophages resembled the M-CSF counterparts, showing a fibroblast-like, elongated shape with lower cell density. In contrast, macrophages in the GM-CSF group were larger, showing more cytoplasmic granules and bigger nuclei (**Figure 3A**). This observation is in accordance with the expression of the pro-inflammatory macrophage markers CD80 and CD86 (23), measured by flow cytometry, the levels of which were considerably higher in GM-CSF-macrophages rather than in M-CSF and AM3-macrophages (**Figure 3B**).

On the other hand, the anti-inflammatory macrophage marker CD163 (23) was expressed higher in M-CSF and AM3 macrophages than GM-CSF counterparts (**Figures 3B, C**). CD68, a general macrophage marker, was used as an internal control to validate the assay. Overall, the phenotype of macrophages differentiated from AM3-stimulated cells resembled that of M-CSF-differentiated (anti-inflammatory) more than GM-CSF-differentiated (pro-inflammatory) macrophages.

### AM3 treatment does not induce innate immune memory in human monocytes

**Figure 4:**
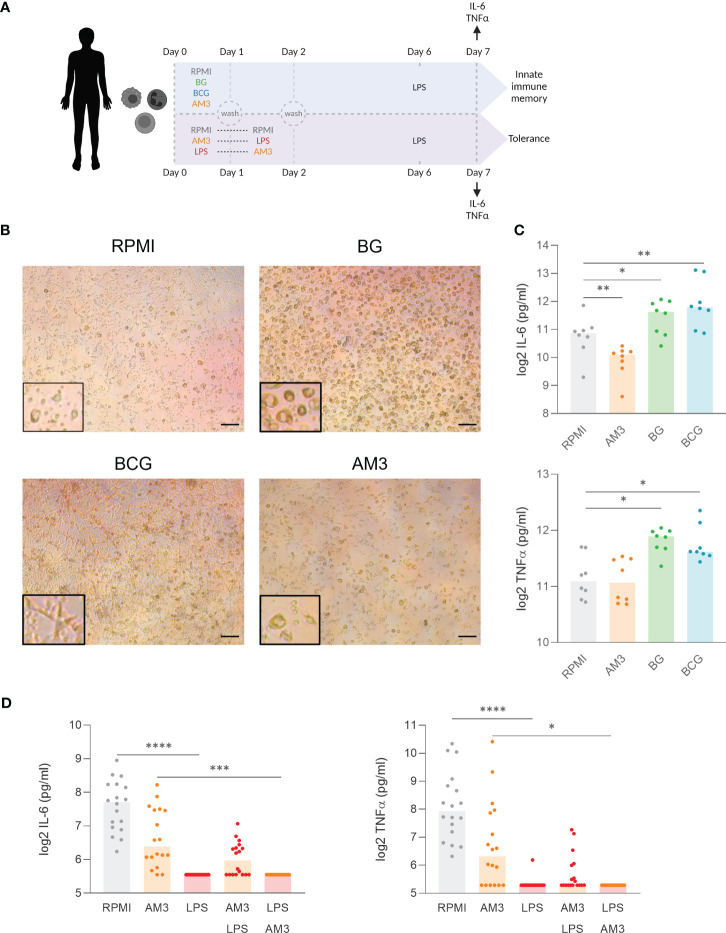
AM3 treatment does not induce innate immune memory in human monocyte. **(A)** Experimental design: First 48 hours were conducted as depicted in **Figure 1A**. Afterwards, adherent cells were left for rest in the base medium supplemented with 10% Human pooled serum for 5 days followed by LPS restimulation. After 24 hours of LPS stimulaion, the supernatants were collected for cytokine measurement. **(B)** Microscope images at day 6 after the first stimulation, magnification:10x, scale bar: 200 µM. The areas highlighted with a black rectangle show an area of the same image with a larger magnification to better appreciate the cell morphology. **(C)** Cytokine production after LPS restimulation at day 7 in the innate immune memory model, n=8, Wilcoxon matched-paired test comparing each condition to RPMI. **(D)** Cytokine production after LPS restimulation at day 7 in the tolerance model. n=18, Friedman test with Dunn’s multiple comparison. *p<0.05, **p<0.01, ***p<0.001, ****p<0.0001.

Given that treatment with AM3 in the first 24 hours skewed the differentiation of macrophages, we sought to elucidate whether AM3 could induce innate immune memory in monocytes using an established protocol (**Figure 4A**, see details in the methods) (17). In these experiments, β-glucan and the live-vaccine BCG, well-established inducers of innate immune memory (13, 24), were used as positive controls.

First, in terms of morphology, monocytes that differentiated in the presence of BG exhibited a prominent, granular phenotype, while cells that underwent differentiation with BCG demonstrated a distinctive elongated star-shape. In contrast, monocytes treated with AM3 remained similar to those cultured in RPMI: small cell bodies with granular appearance (**Figure 4B**). At the level of cytokine production, treatment with AM3 did not induce any increase in the production of cytokines compared to unstimulated cells (**Figure 4C**), independent from the dose used (**Figure S4**). The comparison of the effects in young and elderly volunteers did not show any differences in cytokine production related to age (**Figure S5**). Overall, our study did not reveal any measurable impact of AM3 on the induction of innate immune memory.

It is known that stimulation with LPS induces immune tolerance in the long term (19). In other words, monocytes that produce large amounts of cytokines after acute stimulation with LPS will not produce more cytokines after secondary stimulation. This resembles what happens in humans after an episode of sepsis when there is a strong induction of cytokines in the acute phase, often followed by immune paralysis that can last for weeks or even months (25). Interestingly, in our *in vitro* model, the pre-treatment of monocytes with AM3 prior to LPS stimulation (AM3-LPS group) was able to partially, but not significantly, revert the development of immune tolerance. Supporting this observation, we did not find significant changes in lactate concentration (**Figure S6**), which is an important factor in the metabolic shift in innate immune memory and tolerance (26), throughout the process. We observed that these cells maintained the ability to produce IL-6 and TNFα after secondary challenge with LPS 6 days after the original stimulation (AM3-LPS group), in contrast to cells that were challenged with LPS alone (LPS group), or with AM3 after LPS exposure (LPS-AM3 group) (**Figure 4D**). While the highest concentration of AM3 we used (50 μg/mL) showed a similar effect, lower concentrations had no substantial impact on cytokine production (see **Figure S4**).

## Discussion

An equilibrium between inflammatory and anti-inflammatory factors is required during homeostasis. The immune response must be constantly kept within a homeostatic range to avoid the onset of autoimmune or inflammatory diseases derived from excessive stimulation or the development of immune tolerance with increased susceptibility to infections and cancer (27). In an aging society suffering from the increase in the burden of inflammatory diseases, new strategies for prophylaxis and intervention with safe anti-inflammatory compounds are critically needed. In this study, we have shown the potential of AM3 as a compound to limit inflammatory responses without causing immune tolerance.

When we think of infectious diseases caused by pathogens, we tend to focus on the direct effects of the pathogen on the host. However, the main cause of morbidity and mortality in many infectious diseases is not the direct interaction of the pathogen with our cells but the exacerbated immune response that the infection induces (28). There are multiple examples of this, ranging from bacterial tonsillitis causing throat swelling and fever to cases often involving the patient’s death. For example, the excessive reaction that the immune system mounts to bacteria and fungi is the underlying driver of septic shock (13, 29). Similarly, the many cases of severe COVID-19 with extensive lung damage are due to excessive inflammatory reactions. In these contexts, monoclonal antibodies against IL-6 and IL-1β provide possible therapies to dampen the levels of pro-inflammatory cytokines (30). In our experiments, we observed how pre-treating human cells with AM3 decreases the exacerbated production of cytokines induced by LPS. This property could be employed to prevent cytokine storm response and/or septic shocks in severe infections. Notably, the moderation of cytokine production is not linked with immunosuppression, since cells derived from many individuals maintained cytokine production capacity after exposure to AM3 to levels at least comparable to those of untreated cells. These anti-inflammatory effects align with the capacity of AM3 to skew the differentiation process of monocytes towards a non-proinflammatory phenotype.

In addition to the threat of infectious diseases, we are currently experiencing a silent pandemic of inflammatory diseases: diabetes, gout, rheumatoid arthritis, cardiovascular diseases, inflammatory bowel diseases, autoimmune diseases, and more (31). In this context, using molecules with anti-inflammatory activity can help greatly mitigate the signs, symptoms, and consequences of these chronic diseases (22). All these diseases have in common is the production (either constant or caused by a specific stimulus) of pro-inflammatory cytokines, which trigger a cascade of inflammatory events, increasing myelopoiesis that favors inflammation (32). Our results revealed a marked anti-inflammatory effect of AM3 in human cells suggesting a potential for this compound as a treatment for chronic inflammatory diseases. However, future studies of AM3 applications in specific disease models are needed. The onset and duration of chronic inflammatory diseases have been linked with the induction of maladaptive innate immune memory (31, 33, 34). Unlike other compounds with a fungal origin, such as β-glucan, AM3 did not show the capacity to induce innate immune memory, which supports the safety of its use in the context of inflammatory diseases. The systemic and local production of cytokines by PBMCs and macrophages can help limit disease and control infection spread, in several different conditions, such as tuberculosis, SARS, or influenza (35–38), so the study of compounds with immunomodulatory properties to use as complementary approaches to current therapies or as adjuvants is warranted.

Another intriguing effect observed in this study is the potential of AM3 to prevent the immune tolerance induced by LPS in human monocytes, which is characteristic of people who survive an inflammatory event after bacterial sepsis and causes the death of these patients due to the inability of their immune system to respond to secondary infections days, weeks, or months after the initial infection (39). To our knowledge, only the fungal-derived compound β-glucan has shown the ability to prevent the onset of tolerance (40). The differential potential of β-glucan and AM3 to revert tolerance can be attributed to chemical characteristics. β-glucan, which is known to induce innate immune memory, bears β(1→3) bonds (41). However, different glucose bonds in different glucans from various species can alter their immunomodulatory effects (42). AM3 has β(1→6) and β(1→2), which potentially makes the effects exerted by this compound distinct from those of its close relative β-glucan [β(1→3)]. Another difference is that β-glucan is known to induce long-term activation of the innate immune system, which can be useful in the case of immunocompromised patients but detrimental in those with inflammatory or autoimmune diseases. Thus, our results underline the potential of AM3 as a compound that could help prevent immune tolerance in patients without contributing to inflammation and favoring the maintenance of immune responses within the homeostatic range.

Consequently, we propose that AM3 is a potential candidate for long-term treatment to improve the health status and quality of life of patients with pro-inflammatory diseases. Future clinical trials to assess if AM3 treatment improves the condition and prognosis of patients with chronic inflammatory diseases are needed. Further research to elucidate its potential benefits in inflammatory diseases and the consequences of inflammaging are expected in the upcoming years.

## Data availability statement

The raw data supporting the conclusions of this article will be made available by the authors, without undue reservation.

## Ethics statement

The studies involving humans were approved by MREC Oost-Nederland p/a Radboudumc, house post 628, P.O. box 9101 6500 HB Nijmegen The Netherlands. The studies were conducted in accordance with the local legislation and institutional requirements. The participants provided their written informed consent to participate in this study.

## Author contributions

Conceived and designed the study: JD-A, AR-L and PF-G. Performed the experiments: BG, (human experiments), GK, PD, KF (Documentation for clinical trial, collection, and processing of samples). Analyzed and discussed the data: BG, AR-L, PF-G, and AL. Wrote the paper: BG and JD-A. All authors contributed to the article and approved the submitted version.
