# Supplementary material for: The fungal-derived compound AM3 modulates pro-inflammatory cytokine production and skews the differentiation of human monocytes
Source: Front Immunol. 2023 Oct 9;14:1165683. doi: 10.3389/fimmu.2023.1165683 (PMC10591448; doi:10.3389/fimmu.2023.1165683)
Supplement: Supplementary file 1 [file DataSheet_1.pdf]

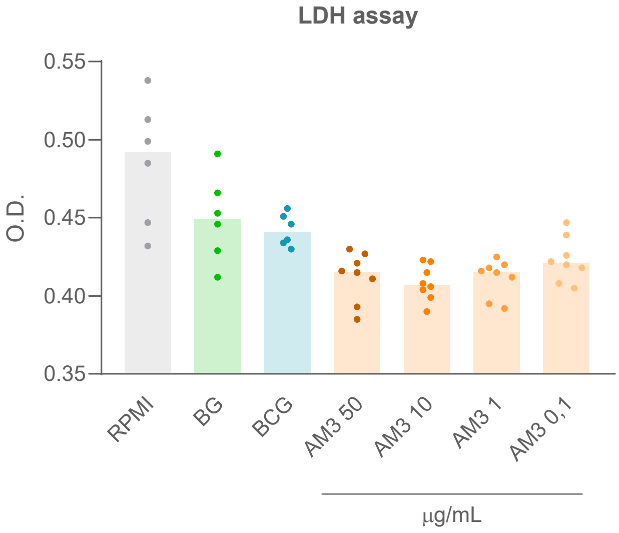

Figure S1. Cytotoxicity of different AM3 concentrations. LDH cytotoxicity assay results from PBMC supernatant after 24-hour stimulation. The stimulation groups are RPMI control, β-glucan (BG, 1 µg/mL) and Bacillus Calmette-Guérin (BCG, 5 µg/mL) and AM3 (50, 10, 1, and 0.1 µg/mL).

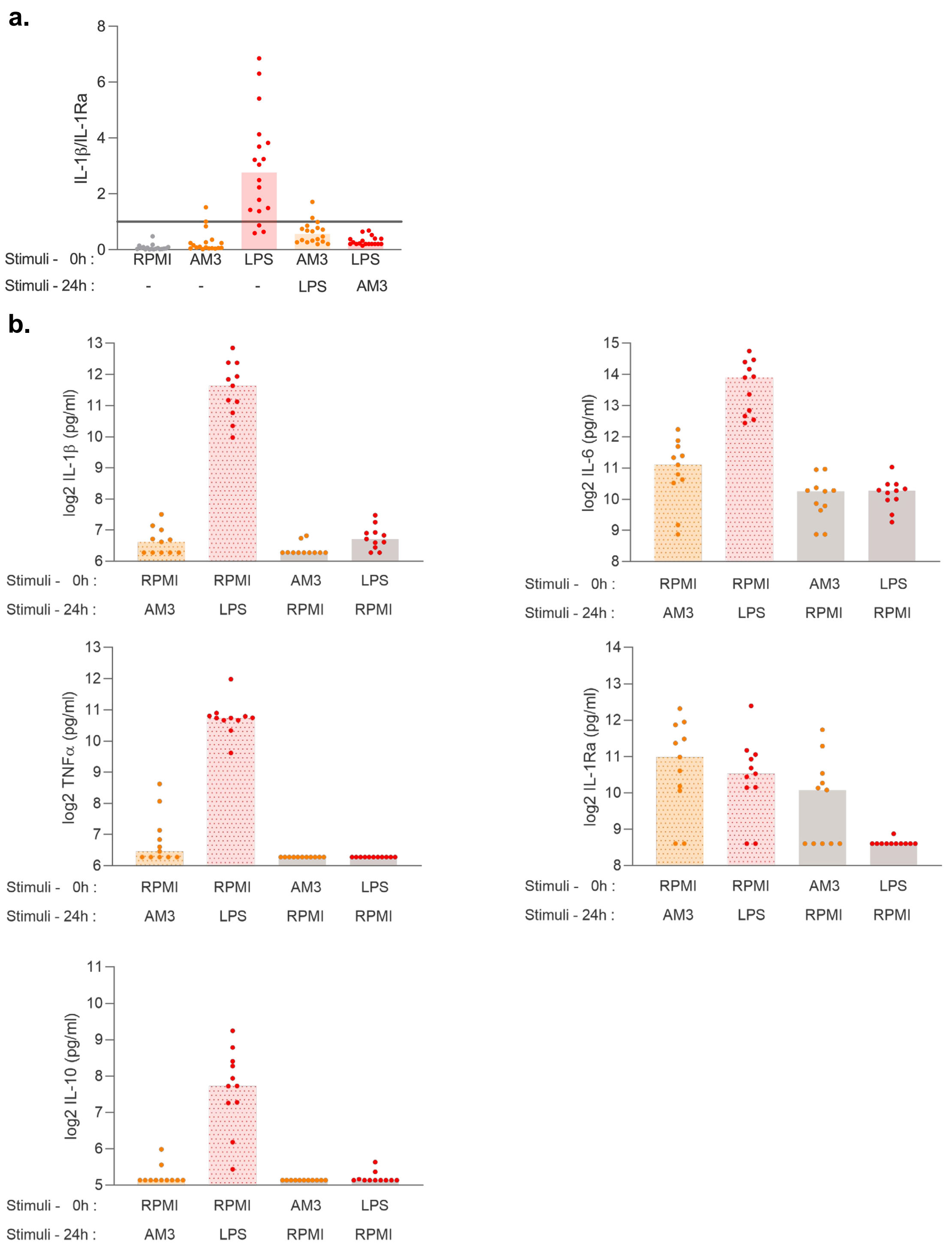

Figure S2. IL-1/IL-1Ra axis and control groups´. (a) Ratio of IL-β/IL-1Ra production from PBMCs at 24 hours and at 48 hours after stimulation with medium control (RPMI), or following alternating prior stimulation with different AM3 concentrations or LPS as depicted on main Figure 1a. The grey line indicates y=1. (b) Cytokine production from PBMCs after stimulation with medium control (RPMI), AM3, or LPS, for 24 hours followed by a washing step, and restimulated with AM3, LPS or medium control (RPMI) for another 24 hours. The cytokine values were therefore measured 48 hours after the first stimulation, as depicted on Figure 1a. n=11 per group.

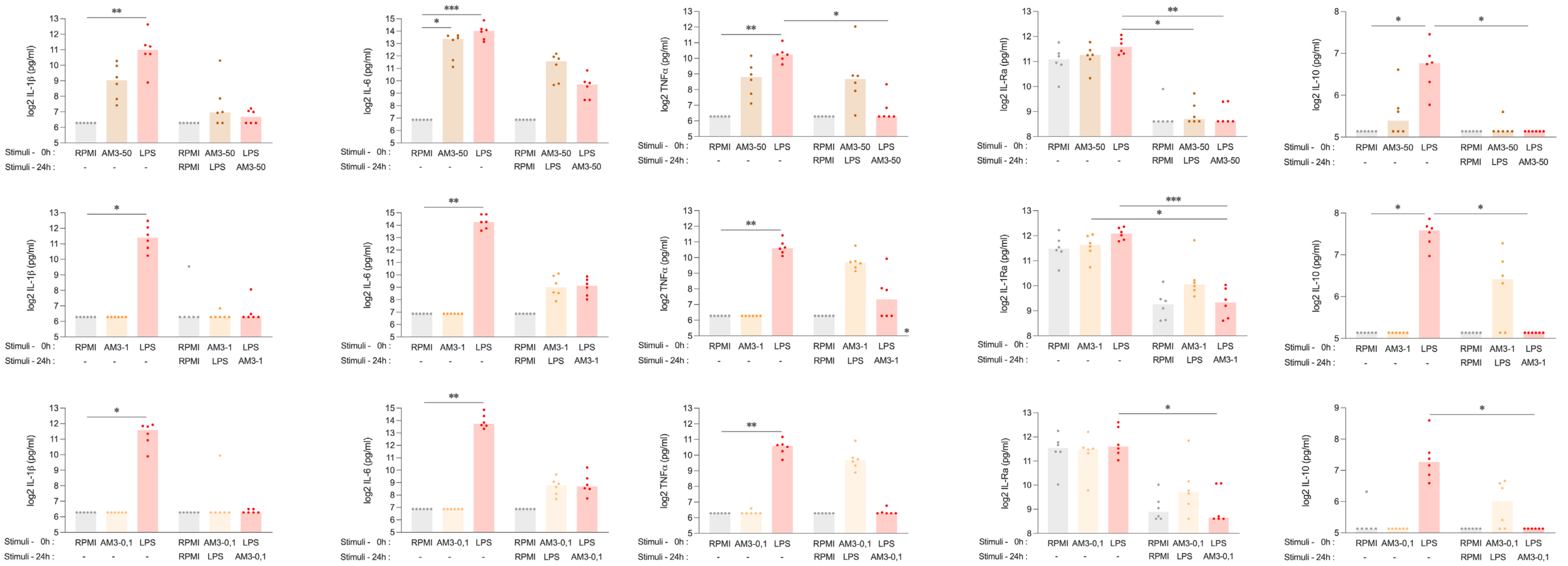

Figure S3. Effect of different AM3 doses on cytokine production from human PBMCs

Cytokine production from PBMCs at 24 hours and at 48 hours after stimulation with medium control (RPMI), or following alternating prior stimulation with different AM3 concentrations or LPS as depicted on main Figure 1a. n=6, Friedman test with Dunn's multiple comparison is applied to compare experimental groups, **: p<0.01, ***: p<0.001, ****: p<0.0001.

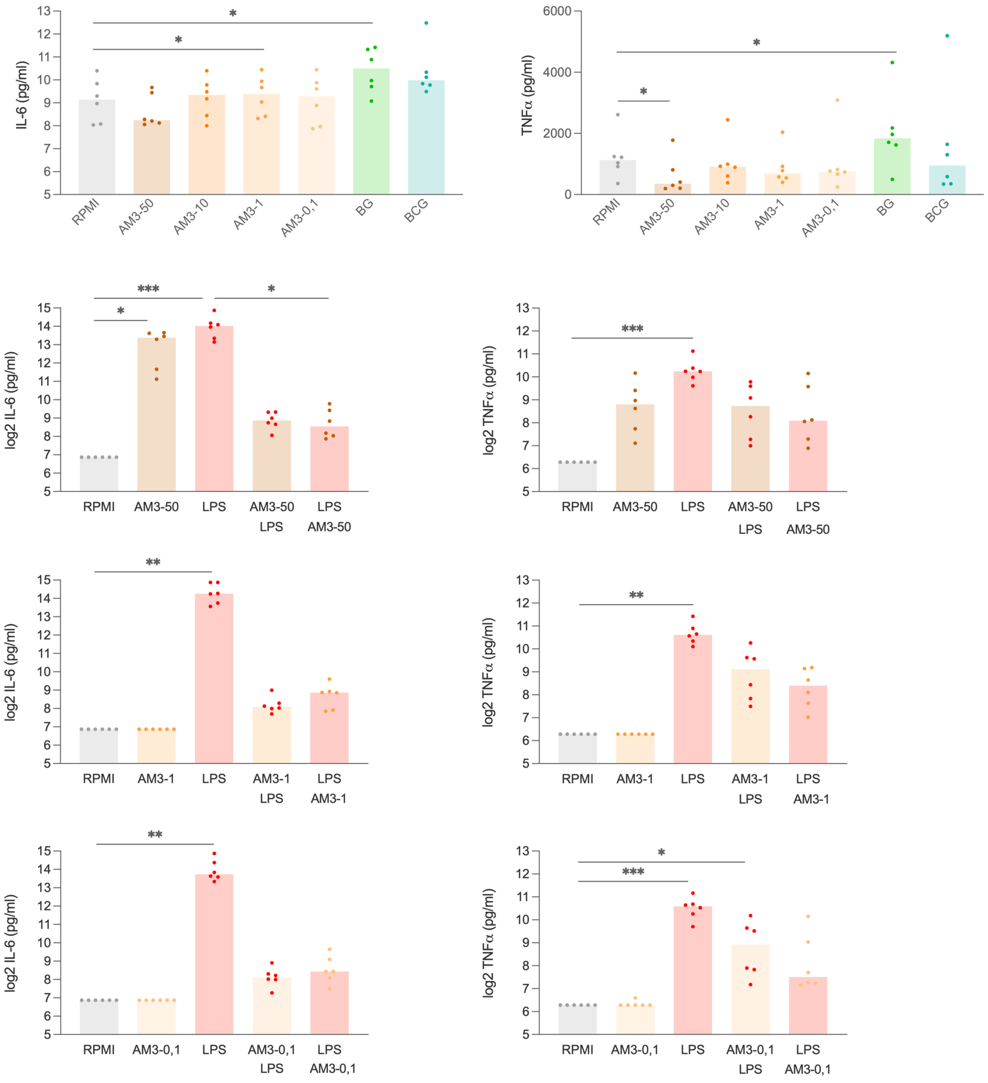

Figure S4. Different doses of AM3 in innate immune memory experiments

(a) Cytokine production after LPS restimulation at day 7 in the innate immune memory model, n=6, Wilcoxon matched-paired test comparing each condition to RPMI (b) Cytokine production after LPS restimulation at day 7 in the tolerance model. n=6, Friedman test with Dunn's multiple comparison. *: p<0.05, ***: p<0.001, ****: p<0.0001.

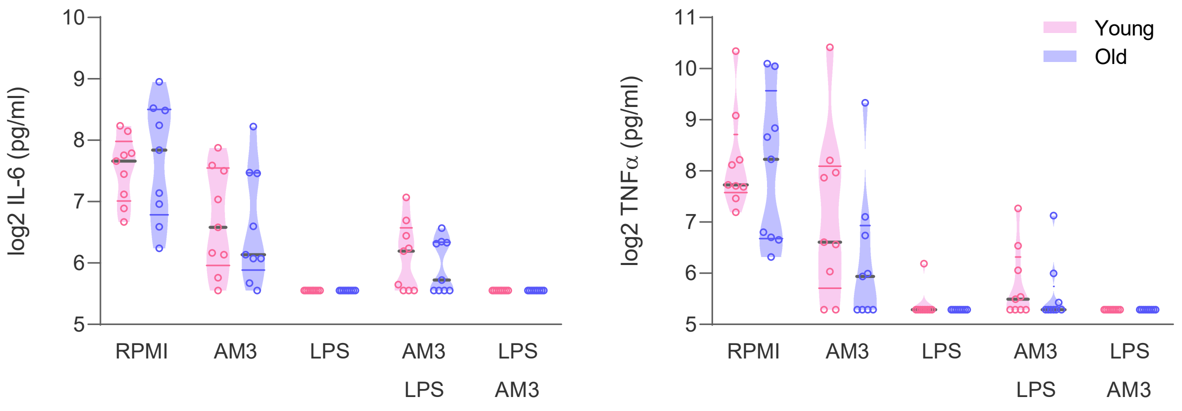

Figure S5. Difference between young and elderly individuals' cytokine production after LPS restimulation in the tolerance model

n=9, Mann-Whitney test used to compare elderly and young indiivuals. Gray line represents the median whereas colored lines represent the borders of the interquartile range.*: $p<0.05$, ***: $p<0.001$, ****: $p<0.0001$.

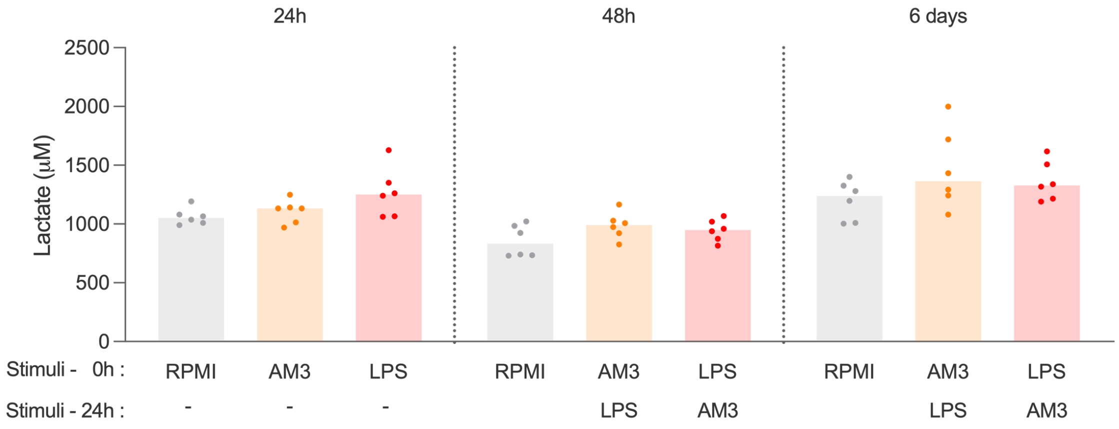

Figure S6. Lactate production throughout time points in the tolerance model

Lactate production at different stages of the tolerance model n=6, Friedman test with Dunn's multiple comparison is applied, and no significance detected.
